# Dipeptidyl Peptidase‐4 Inhibitors Associated Heart Failure Events in Adult Patients With Type‐2 Diabetes Mellitus Treated With Dipeptidyl Peptidase‐4 Inhibitors: A Systematic Review and Meta‐Analysis

**DOI:** 10.1002/edm2.70316

**Published:** 2026-08-19

**Authors:** Shishir Nepal, Shiva Prasad Nepal

**Affiliations:** ^1^ Faculty of Medical Sciences Pokhara Academy of Health Sciences Pokhara Gandaki Nepal; ^2^ Faculty of Management Tribhuvan University Kirtipur Kathmandu Nepal

**Keywords:** dipeptidyl peptidase‐4 inhibitors, DPP‐4 inhibitors, heart failure, linagliptin, saxagliptin, sitagliptin, type‐2 diabetes

## Abstract

**Introduction:**

DPP‐4 inhibitors use has been reported to cause heart failure events in patients with type‐2 diabetes. The FDA had also issued warnings concerning increased risk of heart failure events for alogliptin, saxagliptin and their combination with metformin.

**Methods:**

Randomized controlled trials (RCTs) involving adult patients with type‐2 diabetes patients with or without history of cardio‐vascular disorders were included in the review. Cohort studies, case–control studies and other non‐RCTs, and studies involving paediatric patients were excluded. Data bases searched were PubMed/Medline, DOAJ, MDPI, Google Scholar and clinicaltrails.gov. The search was conducted in late January of 2026. Risk of bias was assessed using RoB‐2 tool for RCTs. Individual study outcomes were tabulated and summary measure was synthesized by meta‐analysis under random effect model; GRADE certainty of evidence was used; sub‐group analysis based on individual DPP‐4 inhibitors, follow‐up duration and placebo or other controls was done; systematic review was done under PRISMA 2020 Guidelines.

**Results:**

The meta‐analysis was done under random effect computation model for all 29 included studies. The analysis for total of pooled 67,873 patients showed statistically insignificant association between the use of DPP‐4 inhibitors and heart failure with Risk Ratio (RR) = 1.06, 95% Confidence Interval (CI) 0.94–1.12. Sub‐group analysis found significant association of saxagliptin use with increased heart failure risk.

**Conclusion:**

Results should be interpreted with caution because of several limitations of the study.

**Trial Registration:**

PROSPERO, CRD420261288081

## Graphical abstract

DPP‐4 Inhibitors associated withheart failure events in patients with type‐2 diabetes. This study found statistically insignificant summary effect for all DPP‐4 inhibitors combined but found asignificant association of saxagliptin with heart failure events (RR = 1.24, 95% CI 1.05–1.47). Results are to be interpreted with caution.

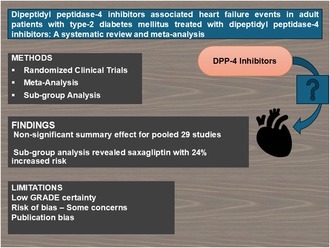

## 1 Introduction

Diabetes mellitus is a clinical syndrome characterized by hyperglycemia caused either by insulin deficiency or absence of insulin completely. Diabetes can be categorized into Type‐1, Type‐2, gestational and diabetes caused by other specific causes called ‘other specific types’ [1, 2]. Autoimmune mediated death of beta cells in the pancreatic islets give rise to syndrome and clinical manifestations of Type‐1 diabetes. Type‐2 diabetes is much more complex, characterized by combination of both improper beta islet cell functions and insulin resistance in muscle and liver tissues [3]. Managing diabetes mellitus aims at maintaining adequate glycemic control via non‐pharmacological interventions through planned diet, weight management, lifestyle modifications and via pharmacological approaches tailored at reducing glycated haemoglobin (HbA1C) level, reducing blood glucose level precisely so as to avoid hyper and hypoglycemia, administering insulin via insulin and insulin analogues and decreasing insulin resistance in muscles, liver and adipose tissues. Ultimately these approaches target at reducing micro and macro vascular complications, keto acidosis, retinopathy, neuropathy and other complications associated with diabetes [4]. Oral antidiabetic agents are effective in managing hyperglycemia in individuals with type‐2 diabetes. These medications encompass sulfonylureas, thiazolidinediones, meglitinide, alpha‐glucosidase inhibitors, GIP (glucose dependent insulinotropic polypeptide) and GLP‐1 (Glucagon‐like peptide‐1) analogues, dipeptidyl‐peptidase‐4 (DPP‐4) inhibitors and sodium‐glucose cotransporter‐2 (SGLT‐2) inhibitors. DPP‐4 inhibitors function by inhibiting the action of dipeptidyl‐peptidase‐4 enzyme of the enterocytes, which rapidly breaks down glucagon‐like peptide‐1 (GLP‐1). GLP‐1 is part of the incretin hormones, which stimulates insulin release in a glucose‐dependent manner, enhancing insulin secretion when blood glucose levels rise. This effect of enhancement of insulin secretion caused by incretin hormones is more pronounced following oral glucose load and is called incretin effect. Furthermore, GLP‐1 decelerates gastric emptying, reduces appetite and supports weight loss, making it suitable for type‐2 diabetes management. By increasing endogenous GLP‐1 levels, DPP‐4 Inhibitors exert their anti‐diabetic effects. Since GLP‐1 promotes insulin release in a glucose‐dependent manner, DPP‐4 Inhibitors and GLP‐1 analogues are less likely to induce hypoglycemia compared to sulfonylureas [5, 6]. DPP‐4 Inhibitors like linagliptin, sitagliptin, saxagliptin, vildagliptin, alogliptin have common side effects of upper respiratory tract infections, nasopharyngitis, headache, arthralgia and urinary tract infections [7]. US Food and Drug Administration's (FDA) review committee had previously issued warnings concerning severe joint aches likely attributed to use of DPP‐4 Inhibitors [8, 9]. The FDA had also issued warnings concerning increased risk of heart failure events for alogliptin, saxagliptin and their combination with metformin. Increased sympathetic activity of cardiac myocytes and potentiation of stromal cell derived growth factor‐1, substance‐P, neuropeptide‐y resulting in myocyte death and ultimately heart failure has been proposed as potential mechanism for these associated heart failure events [10]. Studies have been done on link between increased risk of heart failure events and DPP‐4 Inhibitors use but the results have largely been misinterpreted or were statistically insignificant and inconclusive [11]. Many studies had been done to analyse cardio vascular safety of anti‐diabetic drugs including DPP‐4 inhibitors but very few had been done specifically to address heart failure events. Patients of heart failure with type‐2 diabetes are estimated between 10%–47%, any increase in heart failure risk associated with anti‐diabetic drug like DPP‐4 inhibitors will directly impact such patients [12]. Many previous studies have methodological issues of including cohort, case–control studies and non‐randomized trials. This study aims at addressing this gap by analysing only randomized clinical trials. This study also aims to update the existing literature as many new randomized trials have been done and published in recent years regarding the DPP‐4 inhibitors safety. This study aims at drawing inferences concerning the use of DPP‐4 inhibitors among patients with type‐2 diabetes and risk of heart failure associated with DPP‐4 Inhibitors intake [13]. This study aims to synthesize summary risk‐ratio of heart failure events associated with DPP‐4 Inhibitor use if feasible. As an objective this study will summarize key outcomes of individual included studies in the systematic review.

## 2 Methods

### 2.1 Eligibility Criteria

Studies in the systematic review and meta‐analysis were included prospectively as per the inclusion and exclusion criteria pre‐defined during the protocol registration. Studies were included if they were randomized controlled trials; involving adult and elderly patients aged 18 and above; outcomes as any of heart failure events acute or non‐acute, congestive, hypertensive; and studies reporting the outcome of our concern either primarily or secondarily as listed under adverse events; patients with type‐2 diabetes mellitus with or without any baseline cardiovascular or renal complications. Studies involving paediatric patients, non‐randomized trials, case reports, cohort studies; cross‐sectional studies; systematic reviews, publicly inaccessible studies; studies whose full text was unavailable, studies published in languages other than English were excluded. Duration of follow‐up was not defined strictly, studies reporting the events within the duration of the randomized clinical trial were all included. Clinical trials from clinicaltrials.gov with only published results were included. Unpublished manuscripts, conference abstracts and grey literature were excluded because of limitations of their access, limited resources and time constraints of the authors.

### 2.2 Information Sources

A systematic search was conducted searching for published open access journal articles on PubMed, Google Scholar, MDPI and Directory of Open Access Journals (DOAJ). Clinical trials with published results were also thoroughly searched in clinicaltrials.gov.

### 2.3 Search Strategy

For the systematic search of articles and clinical trials, medical subject headings (MeSH) terms and additional search terms were used; where applicable Boolean connectors (AND, OR, NOT) were also used. Search strings were constructed from following terms: ‘DPP4‐Inhibitor’, ‘gliptin’, ‘heart failure’, ‘type‐2 diabetes’, ‘sitagliptin’, ‘linagliptin’, ‘saxagliptin’, ‘alogliptin’, ‘vildagliptin’. In clinicaltrials.gov, search filters were ticked for ‘Intervention’ and ‘Published‐results’ and following blanks were marked for condition/disease, Type‐2 Diabetes; Other terms, Placebo‐control; Intervention/treatment; ‘Linagliptin, Sitagliptin, Saxagliptin, Alogliptin’; Location was typed as any. Identified and retrieved studies were managed in Zotero version 7.0 for referencing purposes. Document of the complete search strategy is made available publicly and Uniform Resource Locator (URL) of which is given in the end.

### 2.4 Selection Process

Both the authors screened each record; both the authors searched the literature independently and simultaneously; any study missed by either author was reviewed by each other after the initial ‘blinded’ search was completed and those missed studies were later incorporated into the Zotero archive. Search for published journal articles was done on 3 days, 17th–19th January 2026; while clinical trials were searched for 4 days from 19th January to 22nd January 2026.

### 2.5 Data Collection Process

Both authors collected data independently from the final included studies in a single day on 29th January 2026. Relevant data were collected manually and recorded in Microsoft‐Excel as .csv file. Data were later tallied and cross‐checked by each other. In case of any discrepancy, a decision was reached by discussion. Duplicate studies were removed manually. In case of collection of data from studies employing both placebo and regular type‐2 diabetes treatment courses as two separate control groups within the same trial, preference was given to the placebo‐control group.

### 2.6 Data Items

The outcomes of interest were frequency of heart failure events of all types; congestive heart failure, acute heart failure, hypertensive heart failure including number of hospital admissions for heart failure per person as compared to per case and death due to heart failure.

### 2.7 Study Risk of Bias Assessment

Cochrane's Risk of Bias assessment tool (RoB‐2) was used to assess the risk of bias in the included studies. Each of the 5 domains of bias, bias arising from randomization process, due to deviation from intended intervention, due to missing outcome data, in measuring of the outcome and bias in selection of reported result was assessed. For visualization of the results of bias assessment, Robvis was used to construct traffic light map/table.

### 2.8 Effect Measures

The data for outcomes were taken as number of events, then Risk Ratio (RR) was calculated. In case of 0 number of events in any of the elements in 2 by 2 table for RR, Haldane‐Anscombe correction was applied.

### 2.9 Synthesis Methods

Risk for heart failure events was measured using Risk Ratio (RR), Log Risk‐Ratio (lnRR) and variance. Relevant study baseline characters and desired outcomes were tabulated using Microsoft‐Excel software for systematic review and narrative synthesis. A meta‐analysis was done under random effects model with restricted maximum likelihood model using log transformation rule. Log transformed value of summary effect size was converted back to Risk‐Ratio (RR) for interpretation. Meta‐analysis was done using MAJOR package of open‐source software Jamovi ver. 2.7.18. Heterogeneity was analysed using *I* ^2^ statistic. Funnel plot was employed to test the robustness of the study.

### 2.10 Reporting Bias Assessment

Publication bias was assessed using the funnel plot and egger's test.

### 2.11 Certainty Assessment

Certainty of evidence was assessed using GRADE certainty of evidence.

## 3 Results

### 3.1 Study Selection

**FIGURE 1:**
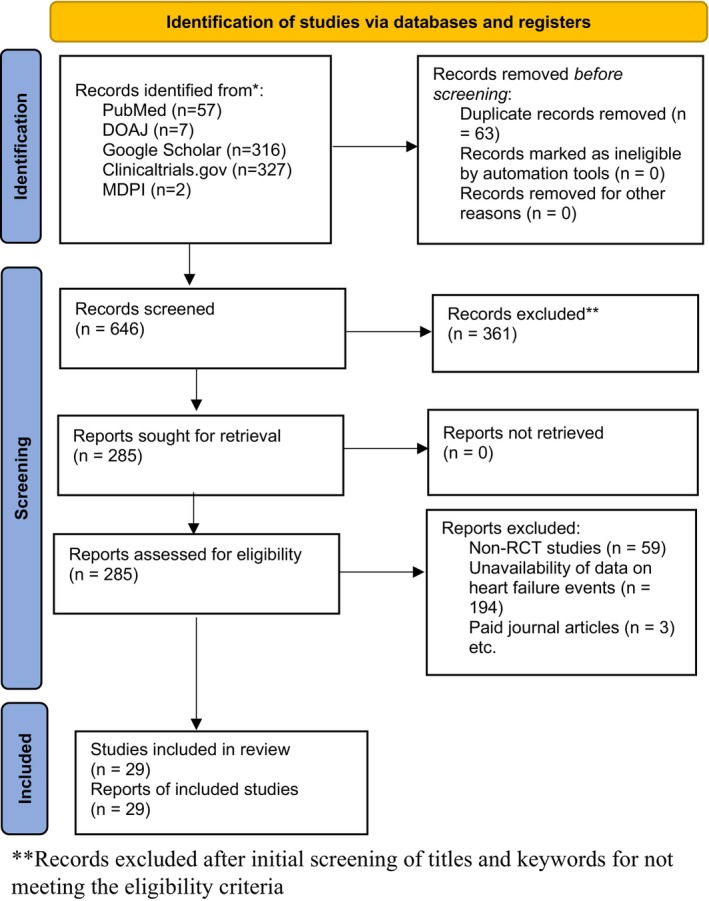
PRISMA flow diagram showing article selection process.

The initial search yielded 709 studies as shown in Figure 1 PRISMA Flow diagram. Sixty‐three studies were duplicates; hence, they were excluded. The remaining studies were screened by their titles and keywords for eligibility, out of which 361 were excluded and 285 were retrieved. Retrieved studies were then assessed for eligibility from their abstracts. Out of 285 retrieved studies, only 29 studies met eligibility criteria and hence were included in the final systematic review and meta‐analysis.

### 3.2 Study Characteristics

Most of the studies were multi‐national (22), 2 studies were done exclusively in the US, 1 in Japan and 1 in China. Location data for 3 studies could not be found in the registry. On the basis of the intervention, 10 studies evaluated Linagliptin, 6 studies evaluated Sitagliptin, 6 studies evaluated Saxagliptin, 4 studies evaluated Alogliptin, 2 studies evaluated Vildagliptin, and 1 study evaluated Omarigliptin. The average age of participants in the experimental group was 61.03 years and in control group was 56.7 years. 16 studies had follow‐up duration more than 6 months, and the remaining 13 studies had follow‐up duration of less than or equal to 6 months. All the included studies were Randomized Clinical Trials. The characteristics of each included study are presented in Table 1.

**TABLE 1:** Characteristics of the included studies.

Studies	Country (% intervention and % in control)	Mean age (years)		Participants		Male%		Study duration	Intervention	Placebo/control	Outcome
		Exp.	Ctrl.	Exp.	Ctrl.	Exp.	Ctrl.				
CARMELINA [14]	27 countries Europe (+South Africa) = 42.16, 41.92 Noth America = 16.98, 16.85 Latin America = 33.08, 33.11 Asia = 7.78, 8.12	66.08	65.65	3494	3485	61.48	64.33	Follow up after 12 weeks, then 24 weeks till end of study (3.5 years), follow up after 30 days since last treatment	Linagliptin 5 mg daily	Placebo	Hospitalization for heart failure
CAROLINA [15]	43 countries Europe = 47,46.5 Noth America, New Zealand or Australia = 20.4, 20.7 Asia = 15.4, 15.5 South America and Mexico = 15, 15.1 Africa (Tunisia and South Africa) = 2.1, 2.2	63.9 ± 9.5	64.2 ± 9.5	3023	3010	60.8	59.2	Follow up of 6.3 years (Median)	Linagliptin 5 mg daily	Glimepiride 1‐4 mg daily	Hospitalization for heart failure
SAVOR‐TIMI [16]	26 countries including USA, UK, India, China, Australia, South Africa and Russia	65.1 ± 8.5	65.0 ± 8.6	8280	8212	66.6	67.3	Follow up of 2.1 years (Median)	Saxagliptin 5 mg daily	Placebo	Hospitalization for heart failure
TECOS [17]	38 countries including USA, UK, India, China, Norway, Russia, South Africa and Australia.	65.4 ± 7.9	65.5 ± 8.0	7257	7266	70.9	70.5	Follow up of 3 years (Median)	Sitagliptin 100 mg daily	Placebo	Hospitalization for heart failure
Gantz et al. [18]	40 countries including USA, Russia, UK, China, Brazil, Denmark, Philippines and Malaysia.	63.7 ± 8.5	63.6 ± 8.5	2092	2100	69.6	70.7	Follow up of 1.8 years (Median)	Omarigliptin 25 mg weekly	Placebo	Hospitalization for heart failure
McMurray et al. [19]	15 countries	62.9 ± 8.5	63.4 ± 10.2	128	126	77.3	76.2	52 weeks	Vildagliptin 50 mg daily	Placebo	Worsening heart failure
NCT02061969 [20]	United States (USA)	68 ± 14	71.5 ± 13	67	73	47.8	34.2	6 months (Total)	Linagliptin 5 mg daily	Insulin glargine 0.1 unit/kg/day	Acute exacerbation of congestive heart failure as adverse event
NCT01794143 [21]	United States (USA)	57.2 ± 10.1	57.0 ± 9.9	1268	1263	62.9	64.2	Follow up Quarterly for 4–7 years	Sitagliptin 100 mg daily	Insulin glargine U‐100 daily	Heart failure as serious adverse event in the trial
NCT01734785 [22]	USA, Australia, Brazil, Spain, El Salvador, Canada, New‐Zealand, France, Norway, South Korea, Spain, Taiwan	55.9 ± 9.6	N/A	606	110	55.5%	N/A	24 weeks	Linagliptin 5 mg daily	Placebo	Congestive heart failure as serious adverse event in the trial
NCT02240680 [23]	USA, Australia, Belgium, Colombia, Denmark, Finland, Germany, Greece, Ireland, Japan, Mexico, New‐Zealand, Poland, Romania, South Africa, Spain, UK	72.3 ± 5.1	72.5 ± 5.6	151	151	60.9	60.3	24 weeks	Linagliptin 5 mg daily	Placebo	Congestive heart failure as serious adverse event in the trial
NCT00954447 [24]	USA, Argentina, Belgium, Brazil, Canada, Czechia, Finland, Germany, Greece, Italy, Mexico, Netherlands, Norway, Peru, Russia, Slovakia, South Korea, Spain, Taiwan	59.7 ± 9.9	60.4 ± 10.0	631	630	52.1	52.2	24 weeks	Linagliptin 5 mg daily	Placebo	Cardiac failure as serious adverse event in the trial
NCT00622284 [25]	USA, Bulgaria, Denmark, France, Germany, Hongkong, Hungary, India, Ireland, Italy, Netherlands, Norway, Poland, South Africa, Sweden, UK	59.8 ± 9.4	59.8 ± 9.4	776	775	59.5	60.8	104 weeks	Linagliptin 5 mg plus metformin	Glimepiride 1‐4 mg plus metformin	Cardiac failure as serious adverse event in the trial
NCT00800683 [26]	USA, Australia, Hongkong, Israel, New Zealand, Ukraine	64 ± 10.9	64.9 ± 9.6	68	65	66.2	53.8	52 weeks	Linagliptin 5 mg daily	Placebo	Heart failure as a serious adverse event in the trial
NCT00798161 [27]	Canada, Croatia, Estonia, France, Germany, India, Lithuania, Mexico, Netherlands, Romania, Russia, Sweden, Tunisia, Ukraine	52.9 ± 10.4	55.7 ± 11.0	143	72	56.9	50.0	24 weeks	Linagliptin 2.5 mg daily plus metformin 1000 mg twice daily	Placebo	Congestive Heart failure as a serious adverse event in the trial
NCT01087502 [28]	USA, Australia, Canada, Finland, Israel, Japan, New Zealand, Slovakia, Sweden	67.3 ± 9.2	65.9 ± 9.4	113	122	61.9	64.8	52 weeks	Linagliptin 5 mg daily	Placebo for 12 weeks then after Glimepiride for 40 weeks	Congestive cardiac failure as a serious adverse event in the trial
NCT00086515 [29]	No location data	54.4 ± 10.4	54.7 ± 9.7	464	237	55.8	59.5	104 weeks	Sitagliptin 100 mg daily	Glipizide 5 mg daily	Cardiac failure as a serious adverse event in the trial
NCT00395343 [30]	No location data	58.3 ± 9.1	57.2 ± 9.3	322	319	48.8	53.0	24 weeks	Sitagliptin 100 mg daily	Placebo	Congestive cardiac failure as a serious adverse event in the trial
NCT00509262 [31]	No location data	64.2 ± 10.7	64.2 ± 9.4	210	212	62.1	57.5	54 weeks	Sitagliptin 25 or 50 mg daily	Glipizide 2.5 to 20 mg daily	Cardiac failure as a serious adverse event in the trial
NCT00127192 [32]	Japan	60.6 ± 7.7	60.2 ± 8.0	68	73	58.8	68.5	12 weeks	Sitagliptin 200 mg daily	Placebo	Cardiac failure chronic as reported under serious adverse event in the trial
NCT02104804 [33]	China	59.3 ± 7.93	58.9 ± 8.17	231	234	47	43.5	24 weeks	Saxagliptin 5 mg daily plus insulin	Placebo plus insulin	Cardiac failure as a serious adverse event in the trial
NCT00614939 [34]	USA, Belarus, Bulgaria, Croatia, Czechia, Estonia, Germany, Hungary, Latvia, Lithuania, Poland, Romania, Russia and Ukraine.	66.8 ± 8.27	66.2 ± 9.08	85	85	37.6	48.2	52 weeks	Saxagliptin 2.5 mg daily	Placebo	Cardiac failure as a serious adverse event in the trial
NCT00121667 [35]	USA, Argentina, Australia, Brazil, Canada, Chile, Mexico, Puerto‐Rico and Taiwan.	54.69 ± 9.62	54.18 ± 10.14	191	179	53.9	53.6	24 weeks	Saxagliptin 5 mg plus metformin	Placebo plus metformin	Congestive cardiac failure as a serious adverse event in the trial
NCT00757588 [36]	USA, Canada, France, Hungary, India, Mexico, Poland, Russia, South Africa and UK.	57.2 ± 9.43	57.3 ± 9.27	304	151	39.5	45.0	52 weeks	Saxagliptin 5 mg plus Insulin daily	Placebo plus insulin	Cardiac failure as a serious adverse event in the trial
NCT00327015 [37]	USA, Argentina, Brazil, Germany, Hungary, India, Italy, Mexico, Philippines, Poland, Puerto Rico, Russia and Ukraine	52.09 ± 10.17	51.83 ± 10.774	335	328	50.4	49.7	24 weeks	Saxagliptin 10 mg	Metformin	Cardiac failure as a serious adverse event in the trial
NCT00968708 (EXAMINE 2013) [38]	50 countries including USA, Australia, Brazil, Canada, India, Japan, Russia, South Africa, UAE, Ukraine and UK	61.0 ± 9.96	60.7 ± 9.88	2701	2679	67.7	68.0	41 months	Alogliptin 25 mg daily	Placebo	Congestive cardiac failure
NCT00286468 [39]	USA, Argentina, Australia, Brazil, Chile, Guatemala, Dominican Republic, India, Mexico, Netherlands, New Zealand, Peru, Poland, South Africa and UK	N/A	N/A	203	99	54.7	51.5	26 weeks	Alogliptin 12.5 mg plus Glyburide	Placebo	Congestive cardiac failure
NCT00286494 [40]	USA, Argentina, Australia, Brazil, Czechia, Germany, Guatemala, Hungary, India, Mexico, Netherlands, New Zealand, Peru and South Africa	N/A	N/A	199	97	55.3	54.6	26 weeks	Alogliptin 12.5 mg daily	Placebo	Congestive cardiac failure
NCT00286442 [41]	USA, Argentina, Australia, Brazil, Chile, Czechia, Germany, Guatemala, Hungary, India, Mexico, Netherlands, New Zealand, Peru, Poland, South Africa and UK	N/A	N/A	207	104	54.3	48.1	26 weeks	Alogliptin 25md daily	Placebo	Congestive cardiac failure
NCT01528254 (VERIFY 2019) [42]	34 countries including Brazil, India, Norway, Russia and South Africa	54.1 ± 9.54	54.6 ± 9.24	998	1001	45.4	48.7	75.6 months	Vildagliptin 50 mg twice daily plus metformin	Placebo plus metformin	Cardiac failure

Abbreviations: N/A, not available, means that the data was unavailable in the registry or record; Exp., Experiment/Intervention group; Ctrl., Control group.

### 3.3 Risk of Bias in Studies

**FIGURE 2:**
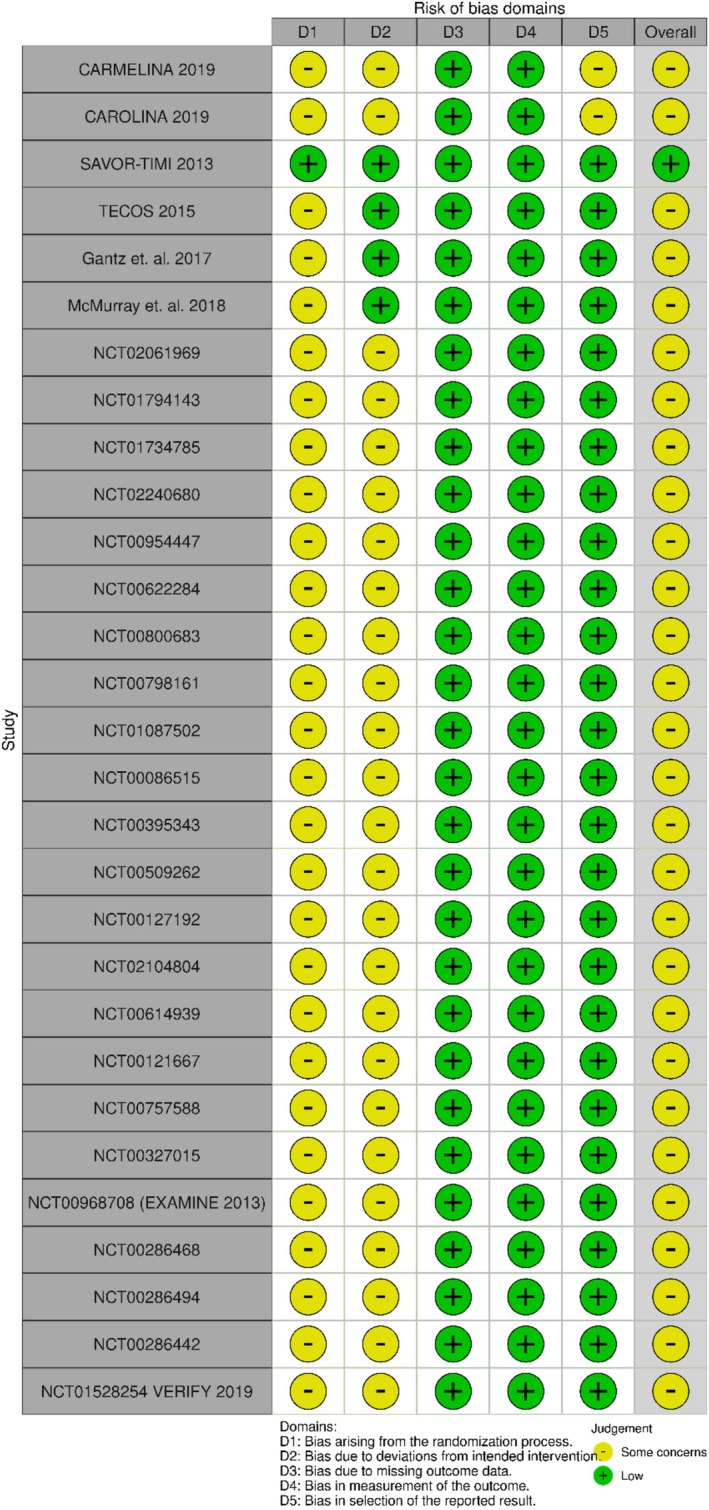
Risk of Bias assessment Traffic Light plot for included studies using RoB‐2 tool and robvis.

Risk of bias for each included study was assessed using the RoB 2.0 Tool and summary of the assessment is present in Figure 2. The figure was generated as traffic light figure using robvis visualization tool [43]. Risk of bias was assessed under 5 different domains, domain D1 concerned with bias arising from randomization process, D2 concerned with bias arising due to deviations from intended intervention, D3 concerned with bias arising due to missing outcome data, D4 concerned with bias in measurement of the outcome and D5 concerned with bias in selection of the reported result. All studies except SAVOUR‐TIMI 2013, had no information regarding the randomization process apart from the simple statement that the study was a randomized trial. SAVOUR‐TIMI 2013 had employed permuted‐block randomization. Thus, there was some concern in domain D1 for 28 of the 29 studies. 4 studies (SAVOR‐TIMI 2013, TECOS 2015, Gantz et al. 2017 and McMurray et al. 2018) had used either intention to treat (IIT) or modified intention to treat (mIIT) analysis to estimate the effect of assignment to intervention but other studies gave no information regarding such analysis thus these 4 studies had low risk in domain D2 while remaining studies had some concern. All studies had low risk of bias in domain D3 and D4. Two studies (CARMELINA 2019 and CAROLINA 2019) had some concern for the domain D5 because no information was available that multiple analysis of the data was done. None of the study had high risk overall bias, majority of study (28 out of 29 total studies) had some concerns of bias, one study (SAVOR‐TIMI 53) had the least risk of bias.

### 3.4 Results of Individual Studies

The results of individual studies are presented in Table 2.

**TABLE 2:** Outcomes for each included study.

Study	Heart failure events in intervention group	Total intervention group	Heart failure in control group	Total control group	Risk ratio	Log risk ratio	Log variance	*p*
CARMELINA [14]	209	3494	226	3485	0.92	−0.08078	0.00863	> 0.05
CAROLINA [15]	112	3023	92	3010	1.21	0.192401	0.01913	> 0.05
SAVOR‐TIMI [16]	289	8280	228	8212	1.26	0.228835	0.0076	> 0.05
TECOS [17]	228	7257	229	7266	1.00	−0.00314	0.008477	> 0.05
Gantz et al. [18]	20	2092	33	2100	0.61	−0.49696	0.07934	> 0.05
Mcmurray et al. [19]	23	128	22	126	1.03	0.028703	0.07318	> 0.05
NCT02061969^a^ [20]	0	67	1	73	0.36	−1.01405	2.6382	> 0.05
NCT01794143 [21]	30	1268	26	1263	1.15	0.13915	0.070214	> 0.05
NCT01734785^a^ [22]	1	606	0	110	0.55	−0.60039	2.655968	> 0.05
NCT02240680 [23]	2	151	1	151	2.00	0.693147	1.486754	> 0.05
NCT00954447 [24]	3	631	2	630	1.50	0.403879	0.830161	> 0.05
NCT00622284 [25]	3	776	2	775	1.50	0.404176	0.83075	> 0.05
NCT00800683 [26]	4	68	1	65	3.82	1.341174	1.2199	> 0.05
NCT00798161^a^ [27]	1	143	0	72	1.52	0.419258	2.645905	> 0.05
NCT01087502 [28]	4	113	5	122	0.86	−0.14651	0.432954	> 0.05
NCT00086515^a^ [29]	1	464	0	237	1.53	0.428846	2.6603	> 0.05
NCT00395343^a^ [30]	0	322	2	319	0.20	−1.61877	2.393769	> 0.05
NCT00509262^a^ [31]	0	210	3	212	0.14	−1.93648	2.276258	> 0.05
NCT00127192^a^ [32]	1	68	0	73	3.22	1.168571	2.638463	> 0.05
NCT02104804^a^ [33]	0	231	1	234	0.34	−1.08576	2.658082	> 0.05
NCT00614939 [34]	1	85	2	85	0.50	−0.69315	1.476471	> 0.05
NCT00121667 [35]	3	191	2	179	1.40	0.340577	0.82254	> 0.05
NCT00757588^a^ [36]	2	304	0	151	2.49	0.913007	2.390115	> 0.05
NCT00327015^a^ [37]	0	335	2	328	0.20	−1.63049	2.393975	> 0.05
NCT00968708 EXAMINE 2013 [38]	63	2701	53	2679	1.18	0.164664	0.033997	> 0.05
NCT00286468^a^ [39]	1	203	0	99	1.47	0.385662	2.651702	> 0.05
NCT00286494^a^ [40]	2	199	0	97	2.45	0.896088	2.384731	> 0.05
NCT00286442^a^ [41]	1	207	0	104	1.51	0.415035	2.652278	> 0.05
NCT01528254 (VERIFY 2019) [43]	3	998	4	1001	0.75	−0.28468	0.581332	> 0.05

^a^ Haldane‐Anscombe Correction has been applied for the calculation of Risk Ratio for studies with 0 events in either intervention or control. The values have been rounded to two decimal places after zero.

### 3.5 Results of Synthesis

29 randomized clinical trials and studies were included in the final meta‐analysis. These studies had population of 34,615 in intervention and 33,258 in control groups. None of the studies had high risk overall bias; the majority of studies (28) had some concerns of bias, one study (SAVOR‐TIMI 53) had the least risk of bias. Linagliptin was administered in most of the studies (10), and one study had used Omarigliptin available outside US as the intervention.

**FIGURE 3:**
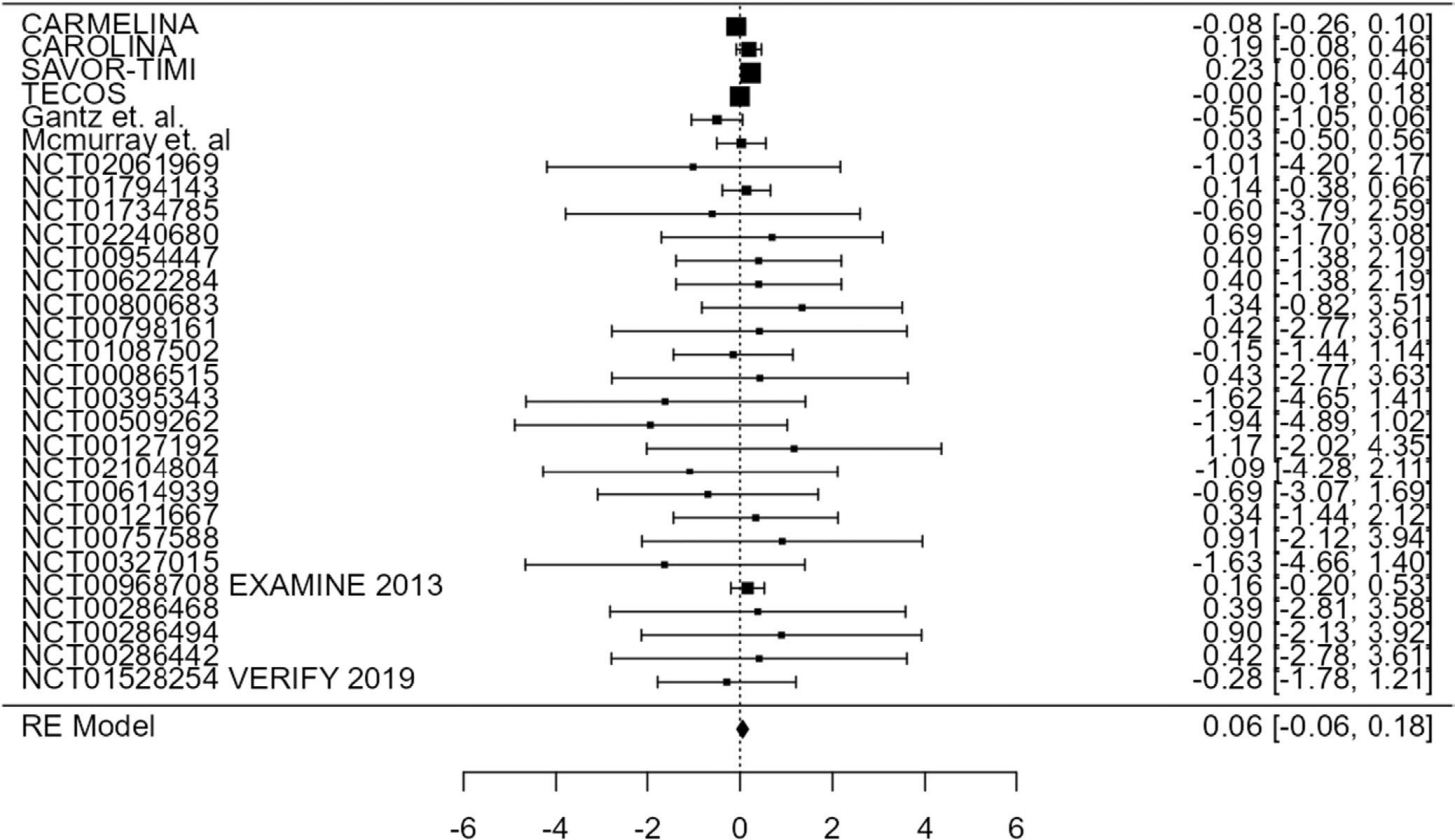
Forest plot of Meta‐Analysis of the 29 included studies under random‐effect model.

The meta‐analysis was done under a random effect computation model for all 29 included studies. The analysis for a total of pooled 67,873 patients showed no statistically significant association between DDP‐4 inhibitors and heart failure, with Risk Ratio (RR) = 1.06, 95% Confidence Interval (CI) 0.94–1.12. The meta‐analysis was done using log transformation rule, and the results were transformed back from log scale to normal scale. The forest plot of the meta‐analysis is presented in Figure 3 which shows summary effect in log transformed value.

Between study heterogeneity was low (*I* ^2^ = 12.82%, *Q* = 20.93, *p* = 0.829), which indicates that the findings of the studies were consistent with each other. Leave‐one‐out sensitivity analysis was done by methodically excluding each individual study included in the meta‐analysis one by one for all 29 studies. When SAVOR‐TIMI 2013 was excluded from the meta‐analysis, the heterogeneity dropped to 0%, and the analysis gave summary effect with RR = 1.00, 95% CI 0.90–1.11. The study that influenced the heterogeneity the most was SAVOR‐TIMI 2013.

**FIGURE 4:**
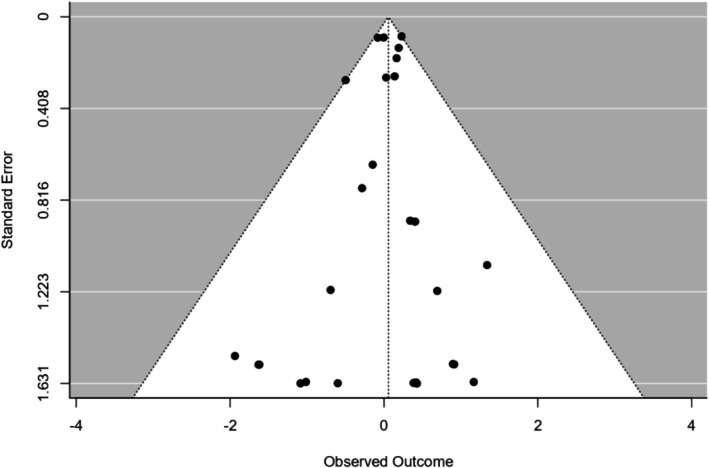
Funnel plot of the observed outcome (Log risk‐ratio) vs Standard error.

Publication bias was assessed using a funnel plot with log risk ratio (Observed outcome) in the X‐axis and standard error in the Y‐axis, Egger's regression and Kendalls Tau. Funnel plot showed no significant signs of asymmetry. Egger's test (*p* = 0.656) and Kendalls Tau (0.616) were not significant. Fail safe N value came 0 because all 29 included studies were statistically insignificant. Overall, there was no evidence of significant publication bias. Funnel plot is presented in Figure 4. Publication bias assessment is presented in Table 3.

**TABLE 3:** Publication bias assessment for meta‐analysis of all 29 studies.

Publication bias assessment		
Test name	Value	*p*
Fail‐Safe N	0.000	0.314
Kendalls Tau	−0.069	0.616
Egger's Regression	−0.446	0.656

*Note:* Fail‐safe N Calculation was done using the Rosenthal Approach.

Certainty of evidence was assessed using the five GRADE considerations (Risk of bias, studies inconsistency, indirectness, imprecision and publication bias) by both the authors independently. Certainty of evidence was assessed as high, moderate, low or very low. As all of the included studies in the meta‐analysis were randomized trials, certainty of evidence started with high. Moderate risk of bias in the included studies dropped the certainty to moderate. Serious imprecision in summary findings dropped the certainty further to low. GRADE certainty assessment is summarized in Table 4 below.

**TABLE 4:** Certainty of evidence assessment by GRADE criteria for meta‐analysis of 29 included studies.

Study/outcome	Certainty assessment					
	Risk of bias	Inconsistency	Indirectness	Imprecision	Publication bias	Overall certainty of evidence
Heart failure event						
29 studies, all Randomized Controlled Trials	Moderate risk of bias	No serious inconsistency	No serious indirectness	Serious imprecision	No significant publication bias	Low ⊕ ⊕ ⊖ ⊖

**FIGURE 5:**
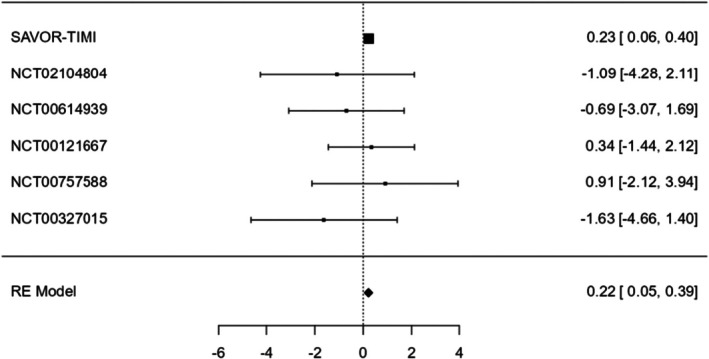
Forest plot of sub‐group studies involving saxagliptin in intervention.

**FIGURE 6:**
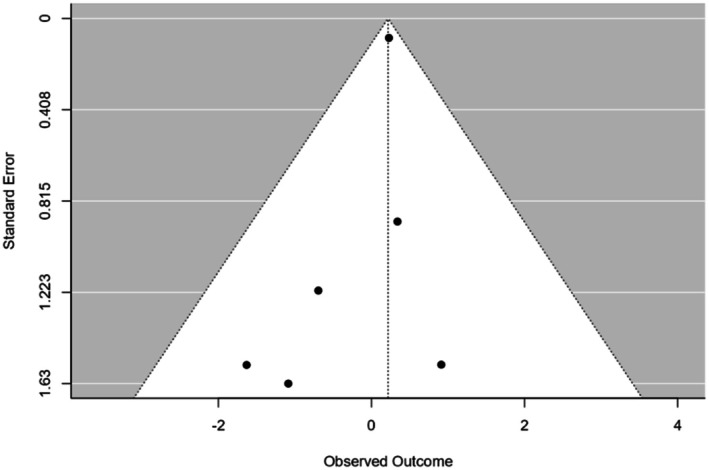
Funnel plot of sub‐group of studies involving saxagliptin in intervention.

Sub‐group analysis was done based on individual DPP‐4 inhibitors. Sub‐group analysis revealed that saxagliptin was associated with statistically significant increased risk of heart failure event, with RR = 1.24, 95% CI 1.05–1.47. Heterogeneity estimated as *I* ^*2*^ statistic was calculated to be 0%. There were no apparent publication bias and egger's test was significant (*p* = 0.325). Forest plot of sub‐group analysis of studies involving saxagliptin as intervention is presented in Figure 5 and funnel plot of sub‐group of studies involving saxagliptin in intervention is presented in Figure 6. Publication bias assessment for sub‐group of studies involving saxagliptin in intervention is presented in Table 5. Sub‐group analysis of studies involving linagliptin as intervention gave statistically insignificant summary effect with RR = 1.05, 95% CI 0.83–1.32. Sub‐group analysis of studies involving sitagliptin as intervention gave statistically insignificant summary effect with RR = 1.01, 95% CI 0.85–1.18. Sub‐group analysis of studies involving alogliptin as intervention gave statistically insignificant summary effect with RR = 1.20, 95% CI 0.84–1.70. Heterogeneity was found to be 0% for both sub‐groups involving sitagliptin and alogliptin as intervention. Heterogeneity for sub‐group of studies involving linagliptin as intervention was found to be 14.21% which dropped to 0% after excluding either of two studies CARMELINA 2019 and CAROLINA 2019. Only 2 studies involved vildagliptins and only 1 study employed Omarigliptin. Sub‐group analysis for these 2 sub‐groups were not feasible.

**TABLE 5:** Publication bias assessment for sub‐group of studies involving saxagliptin in intervention.

Publication bias assessment		
Test name	Value	*p*
Fail‐Safe N	0.000	0.298
Kendalls Tau	−0.600	0.136
Egger's Regression	−0.985	0.325

*Note:* Fail‐safe N Calculation was done using the Rosenthal Approach.

**FIGURE 7:**
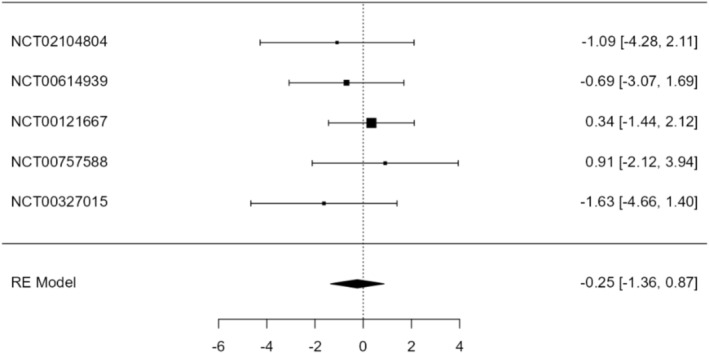
Forest plot of sub‐group of studies involving saxagliptin in intervention omitting SAVOR‐TIMI 2013.

Leave‐one‐out analysis for sub‐group of studies involving saxagliptin as the intervention revealed that majority of thecontribution to the summary effect was from SAVOR‐TIMI 2013. Excluding SAVOR‐TIMI 2013 from the meta‐analysis gave an insignificant summary effect with RR = 0.78, 95% CI 0.26–2.38. Heterogeneity as *I* ^2^ estimate was unchanged and was calculated to be 0%. Forest plot of sub‐group of studies involving saxagliptin in intervention omitting SAVOR‐TIMI 2013 is presented in Figure 7.

Sub‐group analysis for two sub‐groups, one group with placebo in control and next group with another regular anti‐diabetic drug except DPP‐4 Inhibitors was done. Sub‐group of studies employing placebo as control showed statistically insignificant summary effect with RR = 1.04, 95% CI 0.90–1.21 with heterogeneity 22.7%. Sensitivity analysis showed that SAVOR‐TIMI was the study contributing most to the heterogeneity. Sub‐group of studies employing another regular anti‐diabetic drug except DPP‐4 Inhibitors also showed statistically insignificant summary effect with RR = 1.15, 95% CI 0.91–1.43 with heterogeneity of 0%.

Sub‐group analysis was also done on the basis of duration of study. Studies were divided into two sub‐groups, one with study duration of more than 6 months and other with study duration of 6 months or less. Sub‐group analysis of studies with follow up duration less than or equal to 6 months gave statistically insignificant summary effect with RR = 1.04, 95% CI 0.50–2.18 and heterogeneity of 0%. Sub‐group analysis of studies with follow up duration greater than 6 months gave statistically insignificant summary effect with RR = 1.06, 95% CI 0.94–1.20 and heterogeneity of 22.05%. Sensitivity analysis revealed that the study SAVOUR‐TIMI 2013 was major contributor to this heterogeneity, excluding it gave summary effect of RR = 1.00, 95% CI 0.90–1.12 and heterogeneity 0%.

## 4 Discussion

DPP‐4 inhibitors work in diabetes by inhibiting dipeptidyl peptidase‐4 enzyme present inside enterocytes that normally degrade endogenous GIP and GLP‐1 ultimately increasing release of insulin. DPP‐4 enzyme which normally degrades neuropeptide‐y and substance‐P is blocked leading to build up of these peptides in body which increases sympathetic activity and stress on myocardial fibres. Also blocking of DPP‐4 enzymes causes elevation of stromal cell derived growth factor‐1 (SDGF‐1) which promotes irregular myocardial fibrosis and inflammation. Thus, increased sympathetic activity of cardiac myocytes and potentiation of stromal cell derived growth factor‐1, substance‐P, neuropeptide‐y resulting in myocyte death and ultimately heart failure has been proposed as potential mechanism for heart failure events associated with DPP‐4 inhibitors use [10]. Our meta‐analysis of 29 Randomized clinical trials found no statistically significant association of DPP‐4 Inhibitors use and heart failure for all DPP‐4 Inhibitors pooled with summary effect of RR = 1.06, 95% CI 0.94–1.12. This is in line with findings of meta‐analysis of randomized controlled trial by Manucci et al. 2021, which also found no significant association of heart failure with DPP‐4 inhibitor use (MH‐OR = 1.05, 95% CI 0.96, 1.15) [44]. Another meta‐analysis looking for cardiovascular outcomes of DPP‐4 inhibitors in total 157,478 patients with type‐2 diabetes mellitus found similarly non‐significant association of heart failure event with DPP‐4 use with OR = 1.05, 95% CI 0.90–1.23; *p* = 0.55, Liu Dan, Jin Biao, Chen Wei and Yun Peng, 2019 [45]. Our meta‐analysis gave summary effect with low heterogeneity of 12.82%, majority of which was contributed by SAVOR‐TIMI 2013. This indicates that most of the studies shared consistent outcomes to each other. Since certainty of evidence was found to be low by GRADE certainty assessment, results can only be interpreted with low confidence. Although our meta‐analysis yielded non‐significant results this does not definitively establish an absence of drug‐specific risk. Sub‐group analysis revealed that saxagliptin was associated with 24% increased risk of heart failure event, RR = 1.24, 95% CI 1.05–1.47. After excluding the study SAVOR‐TIMI 2013, sub‐group of studies involving saxagliptin as intervention showed insignificant summary effect, which suggests that saxagliptin's association with heart failure was largely contributed by this study. However, since any subgroup analysis requires pre specified hypothesis based on biological rationale and separate dedicated meta‐analysis before any conclusion can be drawn with acceptable confidence, our results from this sub‐group analysis alone can only be realized with minimum confidence.

## 5 Limitations

Our review searched only for published publicly available free access articles, articles published only in English language and excluded paid access articles and grey literatures the conclusions of the study can only be interpreted with caution because of many missing studies. Funnel plots and egger's test has been challenged because they are unreliable for meta‐analysis with low event rates, low number of studies and high false positives or type‐I errors [46]. Because of lack of sufficient resources, funding, lack of access to paid access data‐bases, paid articles, our meta‐analysis couldn't search for wider data‐bases and repositories, and hence our study was limited. Our review looked at only the heart failure events and completely overlooked other micro and macro vascular, cardiac and renal complications as possible adverse outcomes associated with use of DPP‐4 Inhibitors. The studies were assessed by Risk Ratio and quantitative end points of Heart failure events for example, Ejection fraction as function of DPP‐4 inhibitor use was not implicated. Our review included only randomized clinical trials and overlooked evidences by case reports, cohort studies and other cross‐sectional studies. Also, all DPP‐4 inhibitors were pooled together and those with concurrent metformin and insulin addition therapies were also pooled under one meta‐analysis. Publication bias for studies less than 10 in number by funnel plot and egger's test are limited hence sub‐group analyses were limited by publication bias particularly for saxagliptin (*n* = 6).

## 6 Conclusion

This comprehensive systematic review and meta‐analysis showed non‐significant findings on risk of heart failure associated with DPP‐4 inhibitors' use in patients with type‐2 diabetes. Our study comprised a total of pooled 67,873 patients across 29 studies. Sub‐group analysis showed increased risk of heart failure events with saxagliptin use. Results are, however, to be interpreted with caution, given the low certainty of evidence. Our study highlights the need for a large and fully transparent randomized controlled trial with least risk of bias for each of the individual DPP‐4 inhibitors.

## Author Contributions

**Shishir Nepal:** conceptualization, data curation, formal analysis, visualization, writing – original draft, methodology, investigation, writing – review and editing, resources, supervision. **Shiva Prasad Nepal:** data curation, formal analysis, visualization, writing – original draft, methodology, investigation, writing – review and editing, project administration, resources, conceptualization.

## Funding

The authors have nothing to report.

## Disclosure

This systematic review has been registered in the International prospective register of systematic reviews (PROSPERO) under the registration number: CRD420261288081, available from https://www.crd.york.ac.uk/PROSPERO/view/CRD420261288081. The protocol of this systematic review has been published in the PROPSERO available from https://www.crd.york.ac.uk/PROSPEROFILES/4d2ad69fe3972e7a43f1507e6316ea2e.pdf. Complete search strategy of the review is available publicly from https://www.crd.york.ac.uk/PROSPEROFILES/9a8c0c04ae9190267800d443295691b8.pdf. This systematic review was not funded by any sponsor, person, organization or institutions. The reviewers declare no conflicts of interest. The Risk of Bias assessment tool (RoB‐2) for randomized clinical trials by Cochrane used in this systematic review can be found publicly. The visualization tool robvis is also open source, available at https://doi.org/10.1002/jrsm.1411. The meta‐analysis software used in this systematic review, Jamovi, can be found as open‐source software based on R.

## Conflicts of Interest

The authors declare no conflicts of interest.

## Data Availability

The data that support the findings of this study are available in the manuscript itself.
